# Effects of Igneous Intrusion on Microporosity and Gas Adsorption Capacity of Coals in the Haizi Mine, China

**DOI:** 10.1155/2014/976582

**Published:** 2014-02-26

**Authors:** Jingyu Jiang, Yuanping Cheng

**Affiliations:** ^1^Faculty of Safety Engineering, China University of Mining & Technology, Xuzhou, Jiangsu 221116, China; ^2^National Engineering Research Center for Coal & Gas Control, China University of Mining & Technology, Xuzhou 221008, China; ^3^Post-Doctoral Research Station, Yima Mining Corporation Limited, Sanmenxia, Henan 472300, China

## Abstract

This paper describes the effects of igneous intrusions on pore structure and adsorption capacity of the Permian coals in the Huaibei Coalfield, China. Twelve coal samples were obtained at different distances from a ~120 m extremely thick sill. Comparisons were made between unaltered and heat-affected coals using geochemical data, pore-fracture characteristics, and adsorption properties. Thermal alteration occurs down to ~1.3 × sill thickness. Approaching the sill, the vitrinite reflectance (*R*_*o*_) increased from 2.30% to 2.78%, forming devolatilization vacuoles and a fine mosaic texture. Volatile matter (VM) decreased from 17.6% to 10.0% and the moisture decreased from 3.0% to 1.6%. With decreasing distance to the sill, the micropore volumes initially increased from 0.0054 cm^3^/g to a maximum of 0.0146 cm^3^/g and then decreased to 0.0079 cm^3^/g. The results show that the thermal evolution of the sill obviously changed the coal geochemistry and increased the micropore volume and adsorption capacity of heat-affected coal (60–160 m from the sill) compared with the unaltered coals. The trap effect of the sill prevented the high-pressure gas from being released, forming gas pocket. Mining activities near the sill created a low pressure zone leading to the rapid accumulation of methane and gas outbursts in the Haizi Mine.

## 1. Introduction 

Many coalbed methane (CBM) basins such as the Raton and San Juan basins in the USA, the Gunnedah Basin in Australia, and the Qinshui and Fuxin basins in China have undergone contact metamorphism or thermal maturation directly or indirectly related to igneous intrusions [1–3]. The igneous intrusions provide a high-temperature and high-pressure environment for coal seams, which promotes the thermal evolution of the coal seam and speeds up the generation of gas [4]. The effects of localized igneous intrusions on the coal rank, petrology, geochemical, microstructure, and adsorption-desorption characteristics have been studied in numerous papers [5–10]. Two igneous sills in the Gunnedah Basin in Australia had positive effects on the gas content of CBM [2]. More recently, [11] suggested that the heating effect of a dike had enhanced not only the adsorption and porosity of metamorphosed coals but also the gas diffusivity and trap capacities of gas storage. Contrary to the opinion of [6, 11] indicated that the intrusion dikes strongly decreased the coal mesopore, micropore volume, porosity, and surface area that may have negative effects on gas migration in coalbeds adjacent to the dike in the Illinois Basin. The influences of igneous intrusions on coal pores and fractures vary significantly depending on the intrusion patterns, the coal ranks after the intrusion, and the nature of the adjacent formation surrounding the intrusion [10]. However, studies on the influences of localized intrusions on coal pore structure and adsorption capacity are still insufficient.

Igneous intrusions thermally and geochemically alter coal, often causing safety problems for underground coal mines [12]. Coal and gas outburst are dynamic disasters which may result in the projection of fragmented coal rock and rapid release of gases from the working face [13]. Five methane outburst disasters associated with dikes and sills occurred in the coal mines in Highveld coalfield of South Africa in the early 1990s [14]. The enhanced methane storage capacity and lower diffusivity of dike material can partly explain the occurrence of gas pockets encountered in the vicinity of intrusions [11]. Since the early 1980s, fifteen methane outburst disasters were reported to be associated with sills in underground mines in the Huaibei Coalfield of China. Three affected underground mines were the Haizi, Shitai, and Wolonghu Mines [15]. Magmatic activity occurred frequently in the Haizi Mine which has suffered eleven gas outbursts and one water inrush accident under a thick-hard igneous rock [4]. However, the effects of sill intrusions on coal adsorption capacity and their implications for methane outbursts have not been fully understood. The purpose of this paper is to study the effects of localized igneous intrusions on coal microporosity and adsorption capacity and their implications for methane accumulation and gas outburst.

## 2. Geological Background

**Figure 1:**
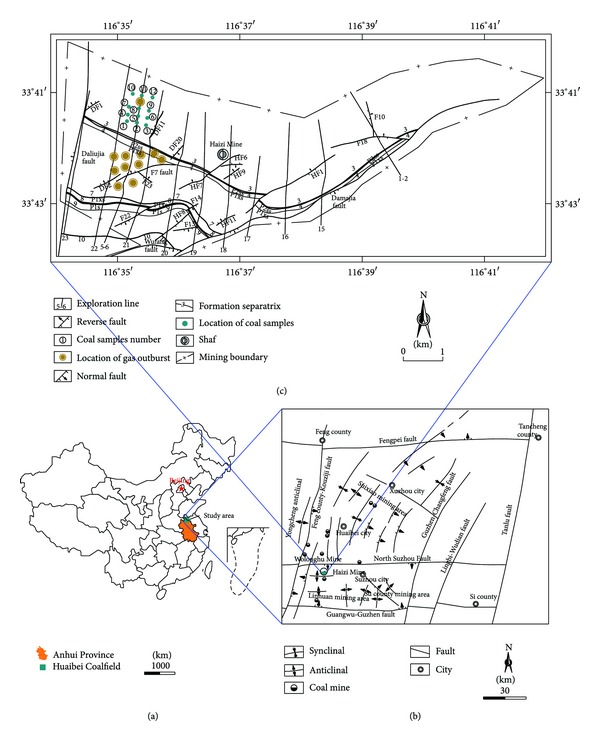
Map showing study area in China (a), geologic structure of the Huaibei Coalfield (b), and data locations of the coals from Haizi Mine (c).

The Huaibei Coalfield, located in the northern Anhui Province in China, has three mining areas: Suixiao, Suxian, and Linhuan. The Huaibei Coalfield is one of the country's major coalfields, with 23 active underground coal mines. The coals of Permian age are used mainly for power generation [16]. The effect of igneous intrusions on coal geochemistry, adsorption capacity, and the CBM generation and accumulation in Huaibei Coalfield was macroscopically investigated [10, 17]. The Haizi Mine, belonging to the Linhuan mining areas, is located in the northwestern Huaibei Coalfield and covers 33.8 km^2^. The number 7, 8, 9, and 10 coal seams are the main mining layers. The locations of the study area are shown in Figure 1.

**Figure 2:**
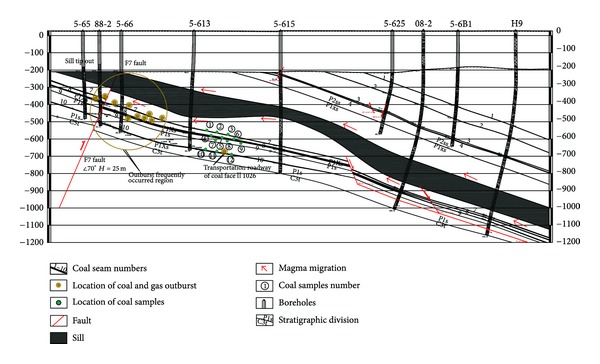
Profile of sill intrusions and coal seams disclosed by boreholes from Haizi Mine.

The Cretaceous Yanshanian magmatic intrusions were widespread in the Haizi Mine, and the number 1 to 10 coal seams were invaded by igneous rock without exception. The diorite sill invades along the number 5 coal seam in the western Haizi Mine, 6.5 km long with a persistent thickness of 120 m [4]. The profile of the extremely thick sill (ETS) and coal seams disclosed by boreholes is shown in Figure 2. The northern part of the Haizi Mine adjoins the North Suzhou Fault which is one of main regional tectonics in the Huaibei Coalfield. The North Suzhou Fault, which is 240 km long and 4–6 km wide, formed in the late Paleozoic coal-forming period [18]. In the Yanshan Period magma upwelled through the North Suzhou Fault, invading the Haizi Mine. The Daliujia and the other faults controlled the direction and distribution of the igneous intrusions (Figure 1).

## 3. Coal Samples and Analytical Procedures

Twelve fresh coal samples were sampled from coal seams number 7, 8, 9, and 10 under the ETS of the Haizi Mine. Three samples from each coal seam were taken at different distances (60–200 m) from the sill (Figure 2). The proximate analysis followed ASTM standards [19]. Random vitrinite reflectance (*R*_*o*_) measurements were performed using a microscope photometer (Zeiss, Germany) according to international standards [20]. The maceral composition of the coals was determined by incident light microscopy and oil immersion according to international standards [21]. Scanning electron microscopy (SEM) analyses were conducted using a HITACHI S-3000N on selected samples. The BET specific surface was obtained using nitrogen gas as the adsorptive at the boiling point temperature of liquid nitrogen, whereas micropore volume, micropore surface area, and average micropore diameter were determined using low-pressure CO_2_ at 273 K (AUTOSORB-1, Quantachrome Instruments Co., USA). We tested the methane adsorption isotherm of dry coal for determining the methane adsorption capacity in coal according to the standard of Chinese coal industry, MT/T752-1997. Crushed coal samples of ~50 g and 0.2–0.25 mm particle size were exposed to gas pressures of up to ~5 MPa at 30°C. The adsorption volume can be obtained using the methods detailed in previous research [22]. The initial gas-releasing rate index, Δ*P*, was obtained according to standards [23]. Gas adsorbed by crushed coal samples of 3.5 g of 0.2–0.25 mm particle size at a pressure of 0.1 MPa was released into a fixed vacuum space. The pressure rise in the space for the period of 10–60 s after the release, Δ*P* (in mmHg), is an index representing the methane release capacity of the coal. The coal firmness coefficient *f* was measured using a dropping hammer according to Chinese coal industry standard, MT 49–1987.

## 4. Results and Discussion 

### 4.1. Coal Rank Analyses

The *R*_*o*_ increases from levels of 2.30% (as measured by oil immersion) to 2.78% (Table 1).

**Figure 3:**
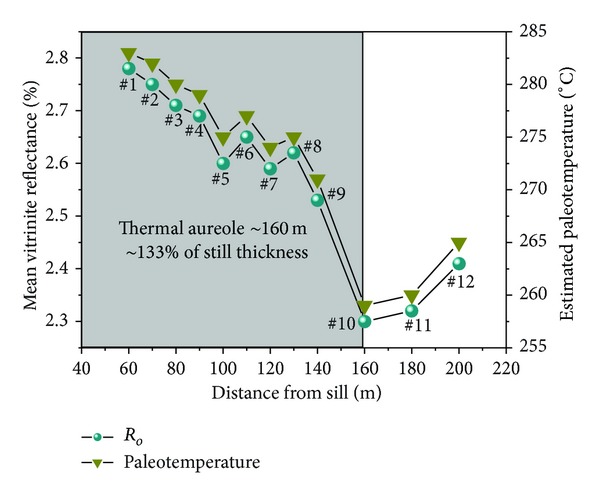
Variation in vitrinite reflectance and estimated paleotemperatures with distance from the sill.

Approaching the sill, the *R*_*o*_ curve shows a slight decrease from coal sample 12 (*R*_*o*_ = 2.41%, ~200 m from the sill) of the number 10 coal seam to sample 10; then, there is a rapid increase from sample 10 (*R*_*o*_ = 2.30%, ~160 m from the sill) to sample 1 (*R*_*o*_ = 2.78%, ~60 m from the sill) of the number 7 coal seam (Figure 3).

These variations in *R*_*o*_ suggest that the thermal aureoles of the sill are approximately 160 m, ~1.33 × sill thickness. The slow growth in *R*_*o*_ from samples 10 to 12 follows the normal burial coalification tracks, the buried depth increases, *R*_*o*_ increases due to the geothermal metamorphism (Figure 3), and we therefore believe that samples 11 (*R*_*o*_ = 2.32%, ~180 m from the sill) and 12 (*R*_*o*_ = 2.41%, ~200 m from the sill) were not within the scope of the thermal aureoles. However, samples 1 to 10 did not follow the normal burial coalification tracks, the buried depth increases, and *R*_*o*_ decreased due to the thermal evolution of the ETS. So we believe that coal samples 1 to 10 (inflection point of *R*_*o*_ curve) were within the scope of the thermal aureoles of the sill (Figure 3).

The paleotemperatures attained by the coal can be estimated from Barker and Pawlewicz's relationship [24]. The estimated temperatures for the Haizi coal data are plotted against the distance from the contact in Figure 3. The thermal evolution temperature (°C) of the Cretaceous Yanshanian magmatic metamorphism to the number 7 coal seam of the Haizi Mine is estimated to be at least 283°C.

**Figure 4:**
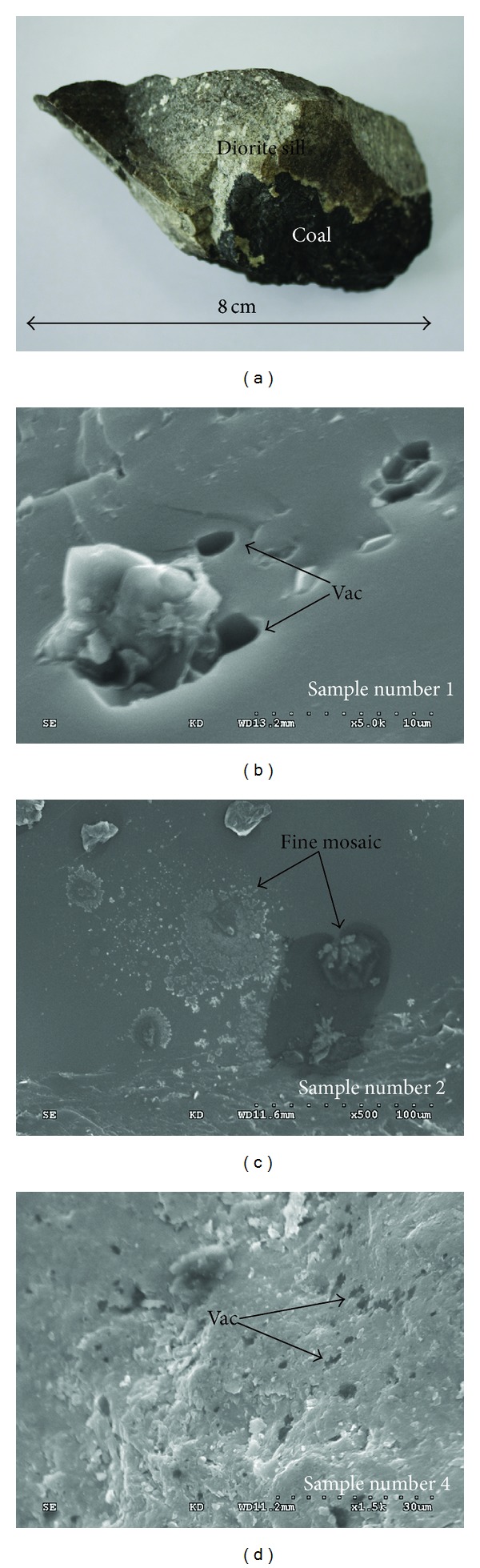
Macroscopic appearance of the igneous sills (a). Photomicrographs (b), (c), and (d) were obtained using SEM. Altered coal showing devolatilization vacuoles (vac), sample 1, *R*_*o*_ = 2.78, magnification 5000x (b); altered coal showing fine mosaic texture, sample 2, *R*_*o*_ = 2.75, 500x (c); altered coal showing (vac), sample 4, *R*_*o*_ = 2.69, 5000x (d).

The macroscopic appearance of the igneous rock by sill intrusion is shown in Figure 4(a). The diorite sill was mostly gray, with some light brown sections. The same megascopic appearance of the sill intrusions was found in the nearby Wolonghu Mine of the Huaibei Coalfield. This result verifies that the diorite rock (approximately 145 Ma), which runs along the North Suzhou Fault [18], was the source of magma both in the Haizi and Wolonghu Mines. Contact metamorphism dramatically increased the rank of the Wolonghu coal (*R*_*o*_ increased from 2.74% to 5.03%) at 0–60 m from the sill in the horizontal direction, while the thermal evolution of the sill in the Haizi Mine slightly increased it in the coal 60–200 m from the sill (vertically) from 2.30% to 2.78%.

Approaching the ETS, devolatilization vacuoles (showing clear indications of thermal alteration) become increasingly prevalent in the heat-affected coals (Figures 4(b) and 4(d)). A fine mosaic texture is seen in sample 2 of the number 7 coal seam closest to the intrusion (Figure 4(c)). The heat-affected coal is dominated by vitrinite (83.4–87.6% by volume), with inertinites ranging between 9.8 and 14.2% and the absence of liptinite macerals (Table 1). However, the unaltered coal is dominated by vitrinite (86.4–88.2% by volume), with inertinites ranging between 8.3% and 9.8%. A similar situation was found in the Zhuzhuang mine of the Huaibei Coalfield; the content of vitrinite decreases with the decreasing distance to the intrusions [10]. Coal maceral and coal rank have major effects on the pore size distribution [25, 26].

### 4.2. Proximate Analysis

**Figure 5:**
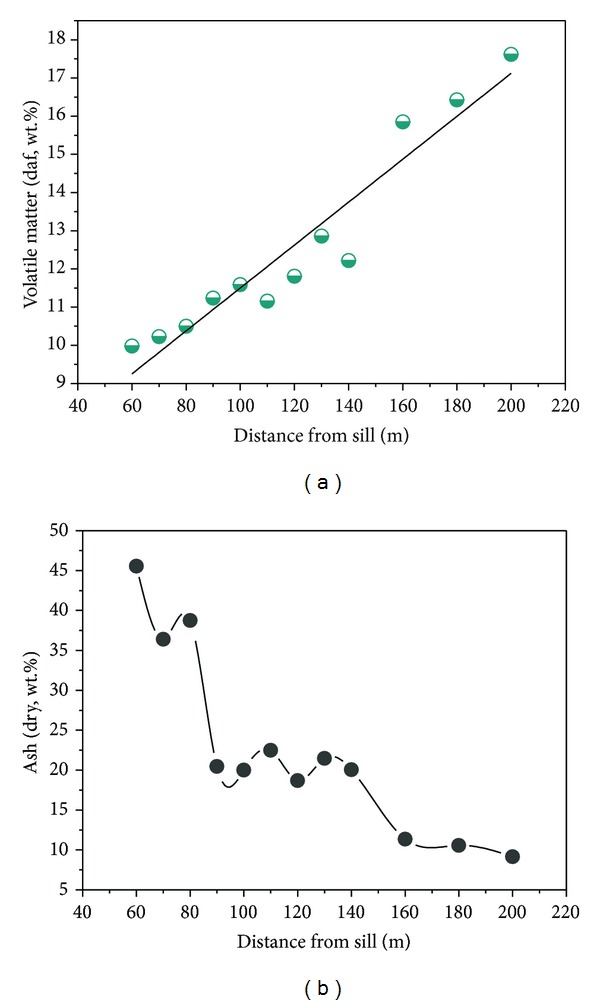
Relation between VM (a) and ash (b) of coal and distance from the sill.

Moisture, ash, and volatile matter (VM) of the samples are listed in Table 1. The sill intrusion affected coal composition (VM, ash, and moisture) out to a distance of ~160 m. The whole-coal VM (daf basis) decreased from 17.6% (sample 12, ~220 m from the sill) in unaltered coal to 10.0% (sample 1, ~60 m from the sill) in altered coal under the sill (Figure 5(a)).

The data are grouped in two distinct groups as a function of the distance. The samples 12, 11, and 10 of the number 10 coal seam which are farthest from the intrusion had high values, reaching a maximum of VM = 17.6% (sample 12, ~220 m from the sill), while the samples 1 to 9 belong to another data group, having a minimum of VM = 10.0% (sample 1, ~60 m from the sill). It is concluded that the thermal evolution of sill decreased the VM and increased the coal rank of coal. Accompanying these trends, the moisture decreased from levels of 3.0% to 1.6% under the sill (Table 1). The ash content (dry basis) increased slightly at first from 9.2% (sample 12) to 22.5% (sample 6); then, there was rapid growth to 45.6% (sample 1) in heat-affected coal near the sill (Figure 5(b)). There was higher ash content in coal samples 3 to 1 (belonging to the number 7 coal seam) near the sill intrusion as the result of contact metamorphism. The number 7 coal seam is just under the sill and is the nearest coal seam to the ETS; magma can intrude directly into the number 7 coal seam along the fault fracture zone (Figure 4(a)).

### 4.3. Pore Size Distribution

#### 4.3.1. BET Surface Area

The BET surface areas of the coal samples obtained by using low-pressure nitrogen adsorptive processes are listed in Table 2.

**Figure 6:**
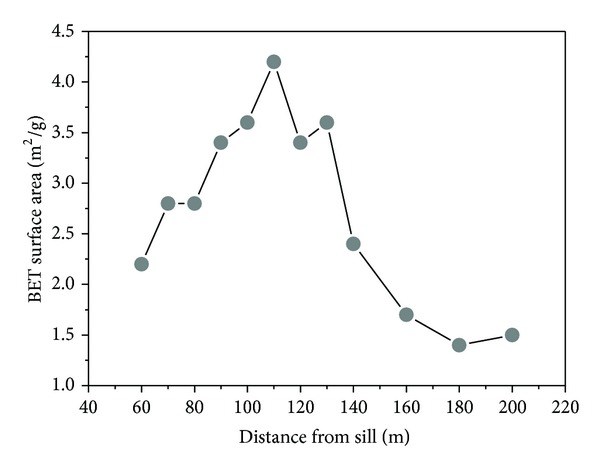
Relation between the BET surface area of coal and distance from sill.

There are obvious differences between samples 1 to 9 and the other samples. Sample 6 (*R*_*o*_ = 2.87%, 110 m from the sill) had a BET surface area of 4.2 m^2^/g, higher than that of other samples. Sample 11 (*R*_*o*_ = 2.44%, 180 m from the sill) had a BET surface area of 1.4 m^2^/g, the lowest value. Approaching the sill, the curve of the BET surface area initially increased and then decreased rapidly close to the ETS (Figure 6). Only for the data of 60–110 m, it seems that the BET surface area decreases close to the intrusion; this may be caused by the coal matrix shrinkage and coal inner heterogeneity when the coal is cooling after the contact metamorphism. The number 7 and 8 coal seams are just under the sill; magma can intrude directly into the coal seams along the fault fracture zone. The trap effect and thermal evolution of the sill obviously increased the BET surface areas of coal (60–160 m from the sill) compared with the coals (160–200 m from the sill).

#### 4.3.2. Microporosity Characteristics

**Figure 7:**
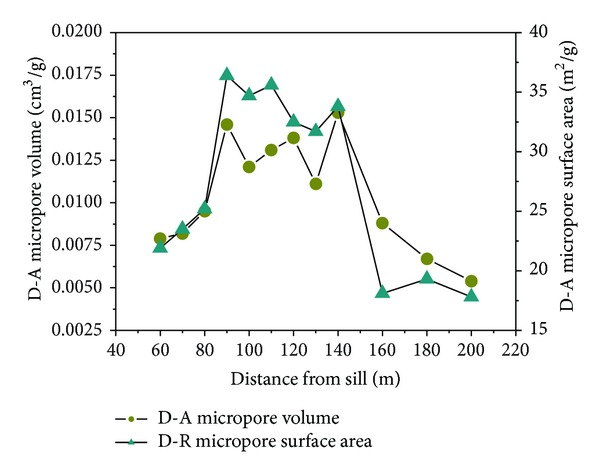
Evolution of micropore volume and surface area with decreasing distance from sill.

The micropore values of coal were obtained using carbon dioxide adsorption, as listed in Table 2. Plotting the Dubinin-Astakhov (D-A) micropore volume and Dubinin-Radushkevitch (D-R) micropore surface area against the distance from the sill, good relativity for the two parameters is shown in Figure 7. The gradients of micropore values are lager near the ETS. This could be due to the contact metamorphism and fluctuations of sill intrusions. In contrast to most coals in other coal-bearing basins in the world, north China coal is well known for its metamorphic complexity resulting from Mesocenozoic igneous intrusions. We simplified the term “multiphasic and superimposed thermal metamorphic evolution” as the fluctuations of igneous intrusions. The uncertainty on microporosity will make the fluctuations of adsorption capacity of Haizi coal.

**Figure 8:**
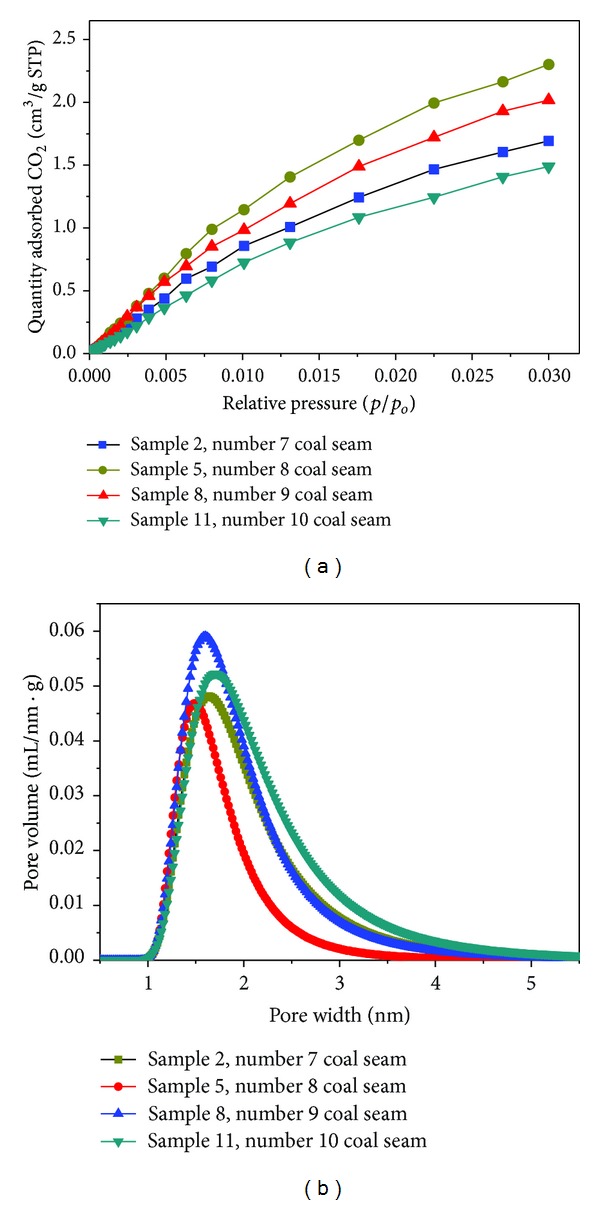
Quantities of carbon dioxide adsorbed onto coals at relative pressure (a) and microspore size distribution as determined by the DA method (b).

The quantities of carbon dioxide adsorbed onto 4 representative samples at relative pressure are shown in Figure 8(a). The pore size distributions as determined by the DA method are shown in Figure 8(b). Note that significantly less CO_2_ is adsorbed onto samples 11 and 2 compared with samples 5 and 8. Overall, the micropore volume on account of CO_2_ adsorption of the coal appears in the following sequence: coal seam number 8 > coal seam number 9 > coal seam number 7 > coal seam number 10 (Figure 8(a)). The heat-affected coal sample 6 (*R*_*o*_ = 2.87%) has the highest average micropore diameter of 1.31 nm, whereas the smallest value of 1.18 nm was found in sample 12 (*R*_*o*_ = 2.41%).

Adsorptive capacity is closely related to micropore (pores < 2 nm) development [25]. Micropore volume is the primary regulator of high-pressure gas adsorption in the Gates coals [27]. The trap effect and thermal evolution of the Yanshan Period sill intrusions obviously increased the micropore volume of coal (60–160 m from the sill) compared with the coals (160–200 m from the sill). This discovery may provide a foundation for understanding the different methane adsorption properties of the Haizi coal samples.

### 4.4. Adsorption and Desorption Properties of Coal

#### 4.4.1. Adsorption Capacity from CH_4_ Isotherms

The Langmuir volume (*V*_*L*_) and Langmuir pressure (*P*_*L*_) of twelve samples from the adsorption CH_4_ isotherms are listed in Table 2.

**Figure 9:**
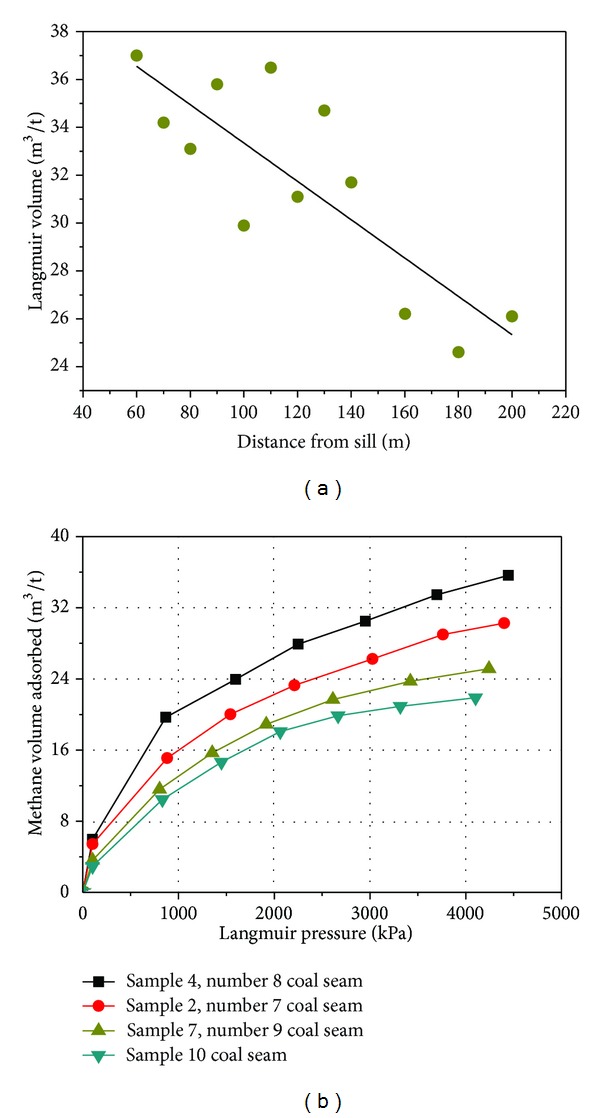
(a) Evolution of the Langmuir volume with decreasing distance from sill and (b) comparison of gas (methane) adsorption behavior of four samples: sample 2, sample 4, and sample 7 in heat-affected zone and sample 10 in unaltered zone.

The Langmuir volume of samples initially increased and then decreased approaching the sill, although with fluctuation (Figure 9(a)). Sample 6 (*R*_*o*_ = 2.65%, 110 m from the sill) has a maximum*V*_*L*_ of 36.5 m^3^/t, while sample 11 (*R*_*o*_ = 2.32%, 180 m from the sill) has a minimum *V*_*L*_ of 24.6 m^3^/t. The adsorption capacity of nine altered samples (samples 1 to 9) has an average *V*_*L*_ of 33.0 m^3^/t which is obviously higher than that of the unaltered coal with an average *V*_*L*_ of 25.6 m^3^/t. The values of the Langmuir volume are of uncertainty. This could be due to the fluctuations of micropore volumes. However, the uncertainty of data creates difficulties for assessment of coal and gas outburst risk. The methane volume adsorbed of four representative coal sample adsorption isotherms shows a regular pattern; the descending order of the coal seams' adsorptive capacity is numbers 8, 7, 9, and 10 (Figure 9(b)).

**Figure 10:**
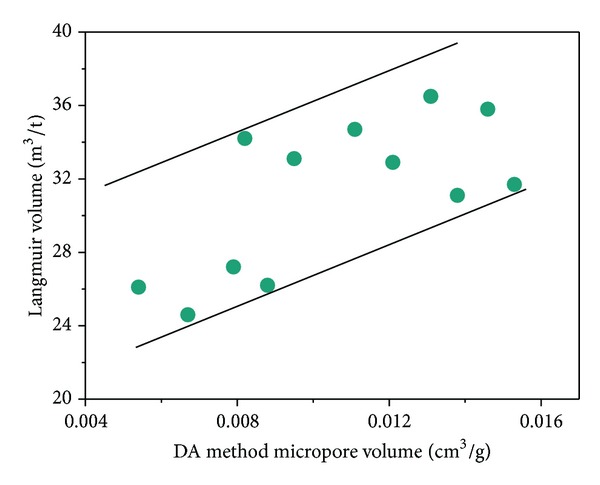
Relationship between the Langmuir volume and the micropore volume as determined by the DA method.

The isothermal adsorption curves of three representative altered samples show that sample 2, sample 4, and sample 7, progressively farther from the sill, have a *V*_*L*_ of 34.2, 35.8, and 31.1 m^3^/t, respectively. These values are much higher than that of the unaltered coal sample 10, sample 11, and sample 12, with an average *V*_*L*_ of 25.6 m^3^/t (Figure 9). The same regularity (adsorption capacity of altered samples is higher than that of the unaltered coal) was found in the igneous intrusion of Zhuzhuang Mine of the Huaibei Coalfield in previous research [10]. The Langmuir volume has an increased trend with the increasing of micropore volume as determined by the DR method (Figure 10).

The linear increase of *V*_*L*_ with the micropore volume indicates that microporosity in coal is the governing factor on *V*_*L*_. The increase of the micropore surface area and the micropore volume enhance the adsorption capacity virtually represented by *V*_*L*_ [22]. Refer to the results in Section 4.1; the thermal evolution of the extremely thick sill obviously increased the coal rank of the Haizi coal under the sill. Coal rank is an important factor affecting the micropore size distribution and thus has an effect on coal adsorption. Approaching the sill, the micropore surface increases with increasing coal rank as does the Langmuir volume.

#### 4.4.2. Methane Initial Gas-Releasing Rate

**Figure 11:**
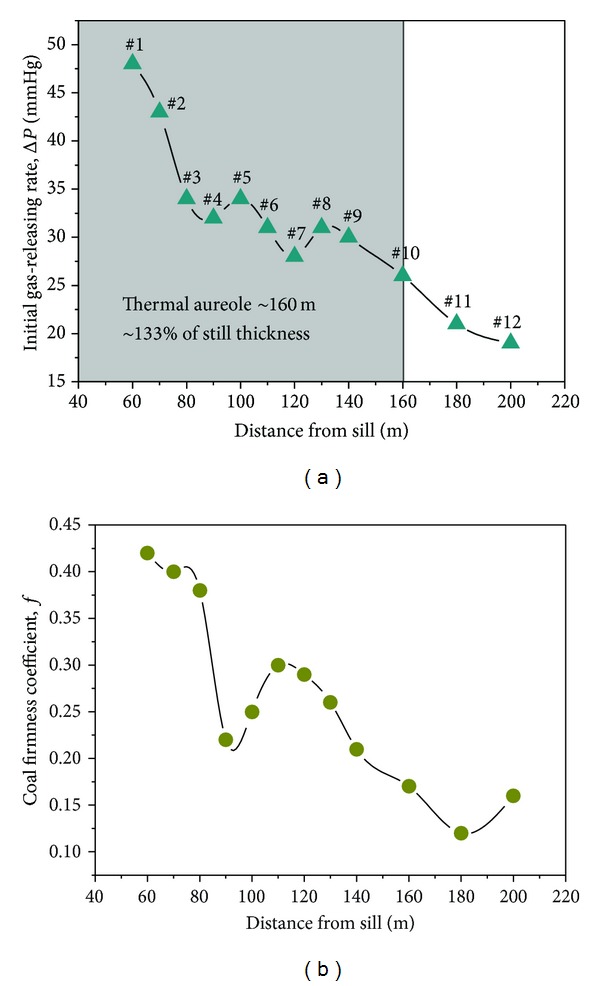
Relation between methane initial gas-releasing rate Δ*P* (a), coal firmness coefficient *f* (b), and distance from sill.

The Δ*P* index [28], based on the initial rate of gas desorption from coal, has been widely adopted in Europe and China. In China, the initial gas-releasing rate, Δ*P*, and the coal firmness coefficient, *f*, of coal from soft layers are considered two of the four indexes for the identification of coal outbursts. The critical value of Δ*P* is 10 mmHg, while the critical value of *f* is 0.5. When the Δ*P* values > 10 mmHg and *f* < 0.5, the coal is considered prone to outbursts. The coal firmness coefficient reflects the physicomechanical properties of the coal seams. The lower the value is, the more easily the outburst occurs. The Δ*P* and *f* measurement results of the twelve coal samples are shown in Table 2. Approaching the sill, both the Δ*P* and *f* of the coal samples increased, reaching a maximum of*f* = 0.42 and Δ*P*_max⁡_ = 48 mmHg, respectively, in sample 1 (Figure 11).

The Δ*P* values of the 12 samples are larger than the critical value, 10 (Figure 11(a)), while its *f* values are lower than the critical value, 0.5 (Figure 11(b)). This result verifies that coal seams number 7, 8, 9, and 10 in the Haizi Mine were coal and gas outburst coal seams. The thermal evolution of the Yanshan Period igneous intrusion obviously increased the gas-releasing rate of the coals that were 60–160 m from the sill compared with the coals that were 160–200 m from the sill. In addition, igneous intrusion substantially altered the physicomechanical properties of the surrounding coal mass. The strength of a coaling machine should thus be increased for cutting in a coal seam with frequent igneous intrusions [29]. The hardness of intrusion and of the mineralized Haizi coal adjacent to the sill can require that companies use hard-rock mining equipment to mine through it.

### 4.5. Effects of Sill Intrusion on Coalbed Methane Accumulation

Coalbed methane was rich in the Haizi Mine, with an absolute emission of 52.1 m^3^/min and a relative emission of 28.2 m^3^/t. A permanent extraction system for coal mine methane exploitation was built in the Haizi Mine, and the extracted methane is used to generate electricity as one of the strategies of greenhouse gas reduction in China [30]. The trap effect and thermal evolution of the sill obviously increased the methane accumulation capacity, the gas pressure, and content of coal seams number 7, 8, and 9 under the ETS. The highest gas pressure observed from number 9 coal seam was 4.50 MPa, with the gas content 17.8 m^3^/t. The biggest gas content obtained from number 8 coal seam was 18.3 m^3^/t, with a gas pressure 2.80 MPa. The gas pressures of the four coal seams were higher than 0.74 MPa, and the gas contents were higher than 8.00 m^3^/t, both higher than the critical values. The gas pressure gradient of number 10 coal seam, in ring-sill trap zone of the Wolonghu Mine, at an elevation above −450 m was 0.003 MPa/m. However, the gradient of elevation below −450 m was 0.027 MPa/m, higher than normal hydrostatic pressure 0.01 MPa/m [15]. This is usually called abnormally high formation pressure [31].

The precise mechanisms of an instantaneous outburst are still unresolved, but the effects of the stress, gas pressure, gas content, and physicomechanical properties of the coal must be considered [32]. Other factors, such as geological features (e.g., igneous intrusions), can combine to exacerbate the problem. The thermal evolution of the Haizi sill obviously increased the capacity of methane generation and retention, while the trap effects of ETS prevented the high-pressure gas from being released. The ETS acted as an impermeable barrier, where pockets of gas have been encountered. Mining activities near the sill would create a low pressure zone, which would allow the adsorptive ability of the enhanced coal to release its gas, leading to localized coalbed methane rapid accumulation and gas outbursts in the Haizi Mine (Figures 1 and 2). Understanding the relationship between outburst disasters and sill intrusions and the mechanism of outbursts, however, requires further rigorous research.

## 5. Conclusions

(1) The Permian-age coal from the Haizi Mine of the Huaibei Coalfield is intruded by Yanshan Period igneous rock. Thermal alteration occurs down to 160 m (~1.33 × sill thickness). Approaching the extremely thick sill, the values of *R*_*o*_ increased from 2.30% to 2.78%, forming devolatilization vacuoles and a fine mosaic texture. Ash (dry basis) increased from 9.2% to 45.6%. The VM (daf) decreased from 17.6% to 10.0%. The moisture decreased from 3.0% to 1.6%.

(2) Approaching the sill, the micropore volumes increased from 0.0054 cm^3^/g (sample 12, 200 m from the sill) to a maximum 0.0146 cm^3^/g (sample 4), followed by a decrease to sample 1 (0.0079 cm^3^/g, 60 m from the sill). The BET surface area initially increased and then decreased rapidly close to the ETS. The Langmuir volume increased from 24.6 m^3^/t in sample 11 (*R*_*o*_ = 2.32%, 180 m from the sill) to 36.5 m^3^/t in sample 1. The methane initial gas-releasing rate Δ*P* increased from 19 mmHg to 48 mmHg.

(3) The thermal evolution of the Yanshan Period sill intrusions obviously increased the micropore volume, the BET surface area, and methane adsorption capacity of the coal 60–160 m from the sill compared with the coal 160–200 m from the sill. This result may provide a foundation for understanding the different methane adsorption properties of unaltered and heat-affected coals of Haizi coals. The ETS acted as an impermeable barrier. The trap effect of ETS prevented the high-pressure gas from being released, forming gas pockets. Mining activities near the sill would create low pressure zone leading to methane rapid accumulation and gas outbursts in the Haizi Mine.

## Figures and Tables

**Table 1:** Geochemical and petrographic analyses of Haizi coal samples.

Sample	CN	*D* (m)	Proximate analysis (wt.%)			Macerals (vol.%)			*R*_*o*_ (%)	*T* (°C)
			Mois	Ash	VM	V	I	M		
Number 1	7	60	1.6	45.6	10.0	83.4	14.2	2.4	2.78	283
Number 2	7	70	1.6	36.4	10.2	85.3	12.7	2.0	2.75	282
Number 3	7	80	1.7	38.8	10.5	85.7	12.0	2.3	2.71	280
Number 4	8	90	2.6	20.5	11.2	87.3	11.3	1.4	2.69	279
Number 5	8	100	2.4	20.0	11.6	87.6	10.5	1.9	2.60	275
Number 6	8	110	2.3	22.5	11.2	87.2	10.8	2.0	2.65	277
Number 7	9	120	2.5	18.7	11.8	86.4	11.2	2.4	2.59	274
Number 8	9	130	2.5	21.5	12.9	87.2	10.6	2.2	2.62	275
Number 9	9	140	2.6	20.1	12.2	85.6	12.0	2.4	2.53	271
Number 10	10	160	3.0	11.4	15.6	86.4	9.8	3.8	2.30	259
Number 11	10	180	2.7	10.6	16.4	87.9	8.8	3.3	2.32	260
Number 12	10	200	2.5	9.2	17.6	88.2	8.3	3.5	2.41	265

Note: CN: coal seam number; *D*: distance from sill boundary; Mois: moisture; Ash is on a dry basis; VM: volatile matter, on dry ash free (daf) basis; V: vitrinite; I: inertinite; M: mineral.

*R*_*o*_: random vitrinite reflectance; *T*: estimated paleotemperature.

**Table 2:** Pore characteristics, adsorptive capacity, and initial gas-releasing rates of coal samples.

Sample	Units	Number 1	Number 2	Number 3	Number 4	Number 5	Number 6	Number 7	Number 8	Number 9	Number 10	Number 11	Number 12
Distance from sill	(m)	60	70	80	90	100	110	120	130	140	160	180	200
BET surface area	(m^2^/g)	2.2	2.8	2.8	3.4	3.6	4.2	3.4	3.6	2.4	1.7	1.4	1.5
D-R micr. surface area	(m^2^/g)	21.9	23.5	25.2	36.4	34.7	35.6	32.5	31.7	33.8	18.1	19.3	17.8
D-A micropore volume	(cm^3^/g)	0.0079	0.0082	0.0095	0.0146	0.0121	0.0131	0.0138	0.0111	0.0153	0.0088	0.0067	0.0054
Avg. micropore size	(nm)	1.21	1.23	1.24	1.33	1.29	1.31	1.26	1.25	1.27	1.19	1.22	1.18
*f*		0.42	0.40	0.38	0.22	0.25	0.30	0.29	0.26	0.21	0.17	0.12	0.16
Δ*P*	(mmHg)	48	43	34	32	34	31	28	31	30	26	21	19
*V*_*L*_	(m^3^/t)	37.0	34.2	33.1	35.8	29.9	36.5	31.1	34.7	31.7	26.2	24.6	26.1
*P*_*L*_	(kPa)	957	808	796	732	1217	822	998	1412	1179	828	1519	812

Note: micropores are pores <2 nm; *f*: coal firmness coefficient; Δ*P*: initial gas-releasing rate; *V*_*L*_: Langmuir volume; *P*_*L*_: Langmuir pressure (daf basis).

## References

[1] Cooper J, Crelling J, Rimmer S, Whittington A (2007). Coal metamorphism by igneous intrusion in the Raton Basin, CO and NM: implications for generation of volatiles. *International Journal of Coal Geology*.

[2] Gurba LW, Weber CR (2001). Effects of igneous intrusions on coalbed methane potential, Gunnedah Basin, Australia. *International Journal of Coal Geology*.

[3] Yao YB, Liu DM, Huang WH (2011). Influences of igneous intrusions on coal rank, coal quality and adsorption capacity in Hongyang, Handan and Huaibei coalfields, North China. *International Journal of Coal Geology*.

[4] Wang L, Cheng YP, Xu C, An FH, Jin K, Zhang XL (2013). The controlling effect of thick-hard igneous rock on pressure relief gas drainage and dynamic disasters in outburst coal seams. *Natural Hazards *.

[5] Dai SF, Ren DY (2007). Effects of magmatic intrusion on mineralogy and geochemistry of coals from the Fengfeng-Handan coalfield, Hebei, China. *Energy & Fuels*.

[6] Mastalerz M, Drobniak A, Schimmelmann A (2009). Changes in optical properties, chemistry, and micropore and mesopore characteristics of bituminous coal at the contact with dikes in the Illinois Basin. *International Journal of Coal Geology*.

[7] Rimmer SM, Yoksoulian LE, Hower JC (2009). Anatomy of an intruded coal, I: effect of contact metamorphism on whole-coal geochemistry, Springfield (No. 5) (Pennsylvanian) coal, Illinois Basin. *International Journal of Coal Geology*.

[8] Schimmelmann A, Mastalerz M, Gao L, Sauer PE, Topalov K (2009). Dike intrusions into bituminous coal, Illinois Basin: H, C, N, O isotopic responses to rapid and brief heating. *Geochimica et Cosmochimica Acta*.

[9] Sarana S, Kar R (2011). Effect of igneous intrusive on coal microconstituents: study from an Indian Gondwana coalfield. *International Journal of Coal Geology*.

[10] Yao YB, Liu DM (2012). Effects of igneous intrusions on coal petrology, pore-fracture and coalbed methane characteristics in Hongyang, Handan and Huaibei coalfields, North China. *International Journal of Coal Geology*.

[11] Golab AN, Carr PF (2004). Changes in geochemistry and mineralogy of thermally altered coal, Upper Hunter Valley, Australia. *International Journal of Coal Geology*.

[12] Saghafi A, Pinetown K, Grobler P, Vanheerden J (2008). CO_2_ storage potential of South African coals and gas entrapment enhancement due to igneous intrusions. *International Journal of Coal Geology*.

[13] He XQ, Chen WX, Nie BS, Zhang M (2010). Classification technique for danger classes of coal and gas outburst in deep coal mines. *Safety Science*.

[14] Anderson SB, Lama R Outbursts of methane gas and associated mining problems experienced at Twistdraai Colliery.

[15] Jiang JY, Cheng YP, Wang L, Li W, Wang L (2011). Petrographic and geochemical effects of sill intrusions on coal and their implications for gas outbursts in the Wolonghu Mine, Huaibei Coalfield, China. *International Journal of Coal Geology*.

[16] Zheng LG, Liu GJ, Wang L, Chou CL (2008). Composition and quality of coals in the Huaibei Coalfield, Anhui, China. *Journal of Geochemical Exploration*.

[17] Liu DM, Yao YB, Tang DZ, Tang SH, Yao C, Huang WH (2009). Coal reservoir characteristics and coalbed methane resource assessment in Huainan and Huaibei coalfields, Southern North China. *International Journal of Coal Geology*.

[18] Han SF (1990). *Coal-Forming Geological Conditions and Forecast of Huainan and Huaibei Coalfield*.

[19] ASTM (2007). Section five, petroleum products, lubricants, and fossil fuels. *Annual Book of ASTM Standards*.

[20] (1994). Methods for the petrographic analysis of bituminous coal and anthracite—part 5: method of determining microscopically the reflectance of vitrinite. *ISO 7404-5*.

[21] Taylor GH, Teichmüller M, Davis A, Diessel CFK, Littke R, Robert P (1984). *Organic Petrology*.

[22] An FH, Cheng YP, Wu DM, Wang L (2013). The effect of small micropores on methane adsorption of coals from Northern China. *Journal of the International Adsorption Society*.

[23] State Administration of Coal Mine Safety of China.

[24] Barker CE, Pawlewicz MJ, Mukhopadhyay PK, Dow WG (1994). Calculation of vitrinite reflectance from thermal histories and peak temperatures: a comparison of methods. *Vitrinite Reflectance as a Maturity Parameter*.

[25] Crosdale PJ, Beamish BB, Valix M (1998). Coalbed methane sorption related to coal composition. *International Journal of Coal Geology*.

[26] Mastalerz M, Drobniak A, Rupp J (2008). Meso-and micropore characteristics of coal lithotypes: implications for CO_2_ adsorption. *Energy & Fuels*.

[27] Clarkson CR, Bustin RM (1999). The effect of pore structure and gas pressure upon the transport properties of coal: a laboratory and modeling study 1. Isotherms and pore volume distributions. *Fuel*.

[28] Ettinger IL, Zhupakhina ES, Schterenberg LE Methods of Allowing Forecasting in the Seams of Coal Zoned Subject to Instantaneous Outbursts.

[29] Singh R, Singh AK, Mandal PK (2002). Cuttability of coal seams with igneous intrusions. *Engineering Geology*.

[30] Cheng YP, Wang L, Zhang XL (2011). Environmental impact of coal mine methane emissions and responding strategies in China. *International Journal of Greenhouse Gas Control*.

[31] Su X, Lin X, Liu S, Zhao M, Song Y (2005). Geology of coalbed methane reservoirs in the Southeast Qinshui Basin of China. *International Journal of Coal Geology*.

[32] Beamish BB, Crosdale PJ (1998). Instantaneous outbursts in underground coal mines: an overview and association with coal type. *International Journal of Coal Geology*.

